# Genome-wide association study of antidepressant treatment resistance in a population-based cohort using health service prescription data and meta-analysis with GENDEP

**DOI:** 10.1038/s41397-019-0067-3

**Published:** 2019-01-31

**Authors:** Eleanor M. Wigmore, Jonathan D. Hafferty, Lynsey S. Hall, David M. Howard, Toni-Kim Clarke, Chiara Fabbri, Cathryn M. Lewis, Rudolf Uher, Lauren B. Navrady, Mark J. Adams, Yanni Zeng, Archie Campbell, Jude Gibson, Pippa A. Thomson, Caroline Hayward, Blair H. Smith, Lynne J. Hocking, Sandosh Padmanabhan, Ian J. Deary, David J. Porteous, Ole Mors, Manuel Mattheisen, Kristin K. Nicodemus, Andrew M. McIntosh

**Affiliations:** 1 Division of Psychiatry, Royal Edinburgh Hospital, University of Edinburgh, EH10 5HF Edinburgh, UK; 2 MRC SGDP Centre, Institute of Psychiatry, Psychology and Neuroscience, King’s College London, London, England; 3 Department of Biomedical and Neuromotor Sciences, University of Bologna, Bologna, Italy; 4 Department of Psychiatry, Dalhousie University, Halifax, NS Canada; 5 Centre for Genomic and Experimental Medicine, Institute of Genetics and Molecular Medicine, Western General Hospital, University of Edinburgh, Edinburgh, UK; 6 Centre for Cognitive Ageing and Cognitive Epidemiology, University of Edinburgh, Edinburgh, UK; 7 MRC Human Genetics Unit, Institute of Genetics and Molecular Medicine, Western General Hospital, University of Edinburgh, Edinburgh, UK; 8 Division of Population Health Sciences, University of Dundee, Dundee, UK; 9 Division of Applied Medicine, University of Aberdeen, Aberdeen, UK; 10 Institute of Cardiovascular and Medical Sciences, University of Glasgow, Glasgow, UK; 11 Department of Psychology, University of Edinburgh, Edinburgh, UK; 12 Psychosis Research Unit, Aarhus University Hospital, Risskov, Denmark; 13 iPSYCH, The Lundbeck Foundation Initiative for Integrative Psychiatric Research, Aarhus, Denmark; 14 Department of Biomedicine and Centre for Integrative Sequencing (iSEQ), Aarhus University, Aarhus, Denmark; 15 Centre for Psychiatry Research, Department of Clinical Neuroscience, Karolinska Institutet, Stockholm, Sweden; 16 Stockholm Health Care Services, Stockholm County Council, Stockholm, Sweden

**Keywords:** Pharmacogenomics, Genetic association study

## Abstract

Antidepressants demonstrate modest response rates in the treatment of major depressive disorder (MDD). Despite previous genome-wide association studies (GWAS) of antidepressant treatment response, the underlying genetic factors are unknown. Using prescription data in a population and family-based cohort (Generation Scotland: Scottish Family Health Study; GS:SFHS), we sought to define a measure of (a) antidepressant treatment resistance and (b) stages of antidepressant resistance by inferring antidepressant switching as non-response to treatment. GWAS were conducted separately for antidepressant treatment resistance in GS:SFHS and the Genome-based Therapeutic Drugs for Depression (GENDEP) study and then meta-analysed (meta-analysis *n* = 4213, cases = 358). For stages of antidepressant resistance, a GWAS on GS:SFHS only was performed (*n* = 3452). Additionally, we conducted gene-set enrichment, polygenic risk scoring (PRS) and genetic correlation analysis. We did not identify any significant loci, genes or gene sets associated with antidepressant treatment resistance or stages of resistance. Significant positive genetic correlations of antidepressant treatment resistance and stages of resistance with neuroticism, psychological distress, schizotypy and mood disorder traits were identified. These findings suggest that larger sample sizes are needed to identify the genetic architecture of antidepressant treatment response, and that population-based observational studies may provide a tractable approach to achieving the necessary statistical power.

## Introduction

Major depressive disorder (MDD) is a disabling condition with a high global impact [1, 2]. Antidepressants are the first-line treatment for MDD patients but response is modest with only approximately 50% achieving remission after completing two treatments [3]. The mechanisms underlying antidepressant resistance remain elusive but are of key value if more effective therapies are to be identified and developed.

Genome-wide association studies (GWAS) of antidepressant treatment response have yet to establish any replicated genetic variants [4–10]. Two large meta-analyses similarly reported no genome-wide significant associated variants. The first, a meta-analysis of the GENDEP (Genome-based Therapeutic Drugs for Depression), MARS (Munich Antidepressant Response Study) and the STAR*D (Sequenced Treatment Alternatives to Relieve Depression) [11] studies comprised of 2256 MDD cases, and the second, between the NEWMEDS (Novel Methods Leading to New Medications in Depression and Schizophrenia) and STAR*D [7] projects comprised of 2897 MDD cases. An additional analysis in the first meta-analysis restricted to citalopram or escitalopram did, however, identify an intergenic variant (5q.15.1) [11]. The largest GWAS to date examining treatment resistance (*n* = 1311) versus responders (*n* = 7795) was conducted by Li et al. utilising self-report information from 23andMe and found no significantly associated genetic variants, although found one variant (4.q22.1) associated with bupropion response [12]. Numerous candidate genes have also been investigated but the results are inconsistent [13]. Furthermore, the largest polygenic risk score (PRS) analysis in antidepressant response to date, (which utilised GENDEP/STAR*D data) yielded no significant associations for response itself, MDD or schizophrenia [14].

Discovering genomic variants associated with resistance to antidepressants could advance personal treatment, help identify resistant individuals earlier and inform our understanding of MDD. A recent systematic review reported non-response was associated with illness severity including higher suicide risk, number of hospitalisations and antidepressant dosage, but not cognitive ability [15]. In fact, several phenotypic associations have been found in treatment resistant individuals; more comorbidities and suicide attempts [16], increased neuroticism and decreased extraversion, openness and conscientiousness [17, 18]. Identifying genetic loci may therefore help to identify resistant individuals earlier and enable timelier intervention.

Currently pharmocogenetic studies are limited by small sample sizes [19] and low statistical power. Numerous studies have indicated the need for large sample sizes in genetic studies [20, 21]. The recent Li et al study maximised sample size by utilising self-report questionnaires [12], whilst other groups have examined treatment resistance in both MDD or schizophrenia by using prescription data [22–24].

In the present study, we employed a complementary approach utilising prescription data in a population and family-based cohort (Generation Scotland: Scottish Family Health Study; GS:SFHS) to define a dichotomous and a semi-quantitative measure of antidepressant resistance; treatment resistance and stages of resistance, respectively. We conducted a GWAS of antidepressant treatment resistance with meta-analysis with the GENDEP cohort and stages of antidepressant resistance in GS:SFHS only and calculated narrow-sense heritability estimates. Gene and gene-set enrichment analysis on both traits were also conducted and we further examined genetic correlations. We also utilised PRS techniques to examine pleiotropy between the genetic liability of MDD, schizophrenia and bipolar disorder in antidepressant treatment resistance and stages of resistance.

## Methods

### Cohort description

#### Generation Scotland: Scottish Family Health Study

GS:SFHS is a family and population-based cohort of 23,960 individuals (mean age = 47.6, s.d. = 15.4) within Scotland. Participants were eligible if they were aged above 18 years and had a first-degree relative also willing to participate in the study. Recruitment has been described in detail elsewhere [25, 26]. Genotype data were available for 20,032 participants and data on mood, cognitive function and personality traits were obtained through interview (see Supplementary Materials). Briefly, four cognitive tests (digit symbol coding, vocabulary, verbal fluency and logical memory), neuroticism and extraversion (measured by the Eysenck Personality Questionnaire), schizotypal personality questionnaire (SPQ), mood disorder questionnaire (MDQ), Scottish Index of Multiple Deprivation (SIMD) and number of years in education were all assessed. MDD was measured by structured clinical interview for DSM-IV (SCID) given a positive screening during the original interview (further details in Supplementary Materials).

Prescription data were available through data linkage to the Prescribing Information System administered by National Health Service (NHS) Scotland Information Services Division. Written informed consent for linkage was obtained for 98% of GS:SFHS and only those individuals that provided informed consent were analysed. Further information regarding the prescription records are found in the Supplementary Materials (see Supplemental Table S1 and S2). To define all MDD antidepressant users, records were excluded if the daily dose was below the minimum recommendations given by the British National Formulary (BNF) for MDD [27] and the duration was below 6 weeks of continuous treatment (as this is considered adequate duration [28, 29]). Following this pruning, we totalled the number of different antidepressants prescribed to each individual. This was then used as a measure of non-response, assuming that switching to a different antidepressant reflected failure or lack of clinical response. Drug switching due to side effects is expected to take place before the 6^th^ week of treatment. Individuals with schizophrenia, schizoaffective disorder and bipolar disorder were excluded (*n* = 164). Additionally, antidepressants per individual were each defined as a “prescription episode” whereby uninterrupted prescriptions for the same antidepressant are considered one prescription episode. This was done in order to differentiate a repeat prescription for one episode from multiple depression episodes where the same antidepressant was given on more than one occasion (more information in the Supplementary Materials).

#### Defining treatment resistance and stages of resistance

Within the antidepressant users (as defined above), treatment resistance was assessed in GS:SFHS using only individuals that had been prescribed at least one antidepressant at an adequate dose and duration (as above, *n* = 3452). Case status for treatment resistance was defined as those individuals who had been prescribed more than two antidepressants providing 250 treatment resistant cases and 3202 non-treatment resistant controls. There have been significant difficulties defining treatment resistant depression in research but the general consensus is that it should be defined as non-response to more than two antidepressants [30].

Individual response to antidepressants decreases with more unsuccessful trials [3], it has therefore been suggested that a semi-quantitative stages of resistance phenotype might be more informative than a dichotomous approach [31]. Stages of antidepressant resistance were defined as the number of different antidepressants prescribed given an adequate dose and duration (as above). It was coded 1–4 with all individuals receiving more than four different antidepressants assigned a value of 4. This definition included 3452 individuals on antidepressants (2557, 645, 186 and 64 on 1, 2, 3 and 4+ antidepressants, respectively). Whilst this definition takes into account the first stage of the Massachusetts General Hospital definition [29], it does not account for augmentation or electroconvulsive therapy (ECT) as these data were not available. This definition therefore provides a semi-quantitative measure of resistance based on the number of treatments taken, providing additional incremental information beyond our binary measure.

#### Genome-based therapeutic drugs for depression

GENDEP is a 12-week study that examined antidepressant response in 867 individuals (mean age = 42.7, s.d. = 11.6) taking escitalopram and noritriptyline. Response was measured by the Montgomery–Åsberg Depression Rating Scale [32]. Antidepressant treatment resistance was defined as those who did not respond to more than 2 antidepressant therapies including GENDEP treatments and previous treatments (cases: 109, controls: 668), as described in a previous study [33]. A full description of the cohort is provided in Supplemental Table S3.

### Genotyping, imputation and quality control procedures

#### Generation Scotland: Scottish Family Health Study

Blood samples were stored and genotyped at the Wellcome Trust Clinical Research Facility, Edinburgh (www.wtcrf.ed.ac.uk). Details of the DNA extraction and genotyping have been given elsewhere [34]. Imputation to a combined reference panel of 1000 Genomes Phase 1 Version 3 and the UK10K haplotype reference panels was completed using Minimac3 and phasing was conducted utilising SHAPEIT2 [35]. All individuals were white British and multidimensional scaling (MDS) components were also used to identify and remove people who were relative outliers for these measures of population ancestry. Quality Control (QC) inclusion criteria were INFO > 0.9, missingness per single nucleotide polymorphism (SNP) or individual < 1%, Hardy-Weinberg equilibrium (HWE) *P*-value cut-off of > 1 × 10^–6^, minor allele frequency (MAF) > 1%. 7,395,460 SNPs and 3452 individuals (and 250 cases for treatment resistance) passed QC criteria.

#### Genome-based therapeutic drugs for depression

DNA was extracted from blood samples and genotyped using the Illumina Human610quad bead chip (Illumina, Inc., San Diego). Imputation to the Haplotype Reference Consortium (HRC) data version 1 reference panel [36] was completed using Minimac3. QC exclusion criteria were poor imputation quality (*r*^2^ < 0.3 (using the Markov Chain method [37])), missingness per SNP > 5%, missingness per individual > 3%, MAF < 1%, related individuals (identity-by-descent > 0.188). Individuals with gender discrepancies, abnormal heterozygosity and population outliers were excluded. 7,518,836 SNPs and 761 individuals (108 cases) passed QC criteria.

### Statistical analysis

#### Bivariate analysis for all antidepressant users and MDD

Bivariate analysis was completed in GCTA v1.91.4 [38] between all antidepressant users (as defined above, *n* = 3452), and Structured Clinical Interview for DSM Disorders (SCID)-diagnosed MDD (details in Supplementary Materials) to assess the degree of shared genetic architecture between these traits. Bivariate values were taken between two variance component measures; genetics (G) and kinship (K), which represent common genetics and pedigree-associated genetics (including rare variants) respectively. The G component is equivalent to methods used to measure SNP heritability and K is an altered G with a pairwise relatedness threshold less than 0.05 set to zero, this technique has been previously published [39]. The model was controlled for age, sex and the first four MDS components fitted to control for population stratification. Statistical significance was estimated using the likelihood-ratio test (LRT).

#### Genome-wide association study

GWAS in GS:SFHS on antidepressant treatment resistance and stages of resistance were completed utilising linear mixed model analysis in GCTA (Genome-wide Complex Trait Analysis) [38]. Age, sex and the first four MDS components were fitted as covariates and, to account for the family structure in GS:SFHS, genetic relationship matrices (GRMs) were fitted as random effects (see Supplementary Materials). To counter the loss of power that is caused by inclusion of a candidate SNP as both a random effect (in the GRM) and a fixed effect, the leave-one-chromosome-out method was utilised [40]. Due to the use of linear mixed models on a binary trait, treatment resistance, Taylor series transformation [41] was used to convert beta and standard error values from the linear scale to the liability scale (see Supplementary Materials).

GWAS in GENDEP was completed on unrelated individuals utilising logistic regression in PLINK [42]. Models were corrected for age, centre, baseline severity and the first four principal components, to control for population stratification.

Meta-analysis between GS:SFHS and GENDEP in antidepressant treatment resistance was completed in METAL [43] with the inverse variance weighted method. A total of 7,120,598 SNPs were in common across both samples.

#### Gene and gene-set enrichment analysis

Gene and gene-set analysis were completed using MAGMA (v1.04) [44] (further details in Supplementary Materials). Individual level data were utilised for analysis of both antidepressant treatment resistance and stages of resistance in GS:SFHS and summary statistics data used for analysis in GENDEP treatment resistance. Antidepressant treatment resistance in GS:SFHS and GENDEP was then meta-analysed in MAGMA using fixed effect meta-analysis. To map SNPs to gene and biologically-meaningful gene sets, SNPs were annotated using NCBI 37.3 and, for the gene-set analysis, gene-annotation files from the Gene Ontology (GO) Consortium (http://geneontology.org/) were taken from the Molecular Signatures Database (MSigDB) v5.2. GO is an inclusive set of 5917 gene pathways that cover a wide variety of functions, including molecular functions, cellular components and biological processes. Gene sets were corrected for multiple testing using the MAGMA default setting correcting for 10,000 permutations.

#### Pedigree-based heritability

Pedigree-based heritability of antidepressant treatment resistance and stages of resistance was calculated in R using MCMCglmm [45]. This was achieved by constructing a variance component matrix that takes into account all pedigree information and then fitting it into a univariate model as a random effect. MCMCglmm uses a Bayesian framework to estimate heritability. For treatment resistance, the logit link function was used to account for the binary nature of the phenotype.

#### Genetic correlation analysis

Genetic correlations were calculated using a bivariate analysis in ASReml-R (http://www.vsni.co.uk/software/asreml/). Correlations between antidepressant treatment resistance and stages of resistance were examined with eight personality and cognitive variables; neuroticism, extraversion, schizotypal personality questionnaire (SPQ), mood disorder questionnaire (MDQ), general cognitive ability (‘g’, formed from four varied cognitive test scores), Scottish Index of Multiple Deprivation (SIMD), education and the general health questionnaire (GHQ). The ASReml-R method was utilised as it can account for the family structure in GS:SFHS. Genetic correlation measurements were calculated between pedigree-based heritabilities as the sample sizes were too small to conduct SNP-based correlations. More information on the variables and methods used can be found in the Supplementary Materials.

#### Polygenic risk scoring analysis

PRS were constructed utilising PLINK [42]. This method has been previously described [46] and further information is available in the Supplementary Materials. Summary statistics taken from the Psychiatric Genomics Consortium (PGC) were used to construct PRS for MDD (unpublished data, see Supplementary Materials), schizophrenia [47] and bipolar disorder [48] in the GS:SFHS cohort to examine genetic liability to the disorders in a treatment resistant population. PRS were reported across five *P*-value thresholds (<0.01, <0.05, <0.1, <0.5 and <1).

Association of PRS to the trait was analysed by linear mixed model analysis in ASReml-R (http://www.vsni.co.uk/software/asreml/) with antidepressant treatment resistance or stages of resistance as the dependent variable and PRS as the independent variable. All models were adjusted for age, sex, and the first four MDS components and, to account for related individuals, an additive relationship matrix (expected relatedness derived from pedigree information) was fitted as a random effect. Wald’s conditional *F*-test was used to derive *P*-values for all fixed effects (see Supplementary Materials). Taylor series approximation [41] was used for the treatment resistance variable, as above.

AVENGEME [49] was used to calculate power in the PRS analysis assuming 5% of SNPs had an effect in the training sample and all markers were independent. Two theoretical covariances were tested at 0.5 and 0.25.

## Results

### Genetic correlation of all antidepressant users and MDD

Antidepressant use and MDD demonstrated significantly overlapping genetic architectures for both common (*r*_g_ = 1.0, *P* = 0.026) and pedigree-related genetics (*r*_k_ = 0.88, *P* = 1.7 × 10^−16^, Supplemental Table S4). Both correlations indicate that ‘any antidepressant use’ defined from prescription records using dose and duration filters is a valid means of identifying a genetically representative MDD population. Common genetics accounted for 0.10 (CI= 0.02 – 0.19) of the variance in antidepressant use (i.e. SNP heritability) and pedigree-related genetics 0.43 (CI= 0.32 – 0.54), SCID MDD measurements in this cohort have been previously reported (Zeng et al, [50], PMID= 27838479).

### Genome-wide association study

**Fig. 1:**
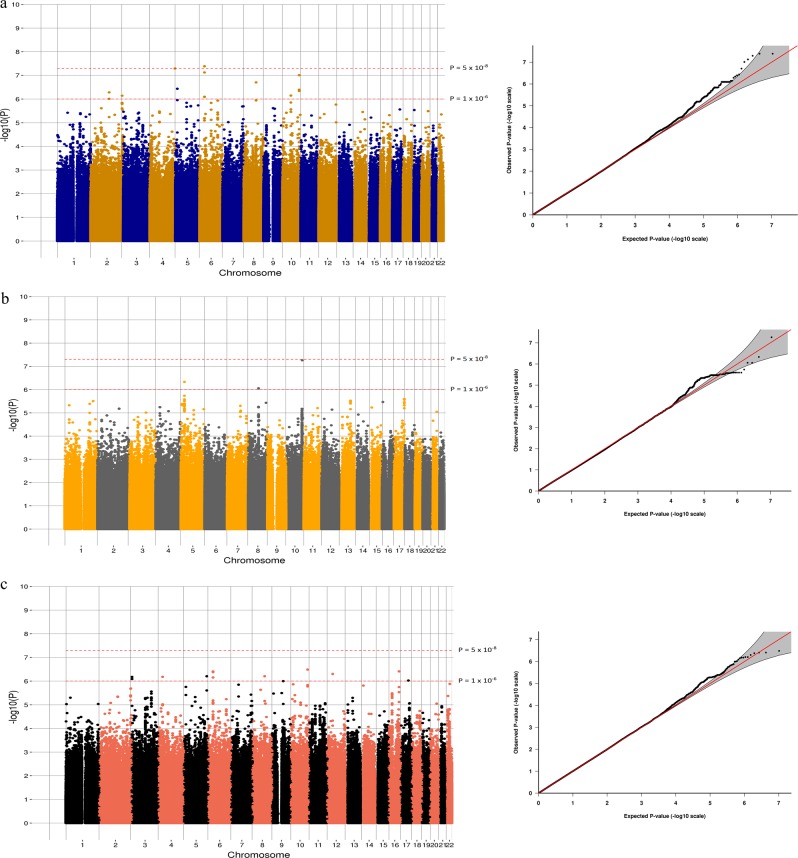
Manhattan and Q-Q plots of the GWAS of antidepressant treatment resistance in (**a**) Generation Scotland: Scottish Family Health Study, (**b**) Genome-based Therapeutic Drugs for Depression and (**c**) the meta-analysis between the two cohorts. Genome-wide significance level (*P* < 5 × 10^−8^) is represented by a red line and suggestive threshold (*P* < 1 × 10^-5^) is represented by a blue line

In the antidepressant treatment resistance meta-analysis of 4213 individuals (cases = 358, controls = 3855), no SNP reached genome-wide significance (*P* > 5 × 10^−8^). The most significant SNP identified was an intergenic variant located at 10p26.13 (lead SNP rs188352979, *P* = 3.25 × 10^−7^, OR = 2.87, CI = 2.47–3.28; Fig. 1).

**Fig. 2:**
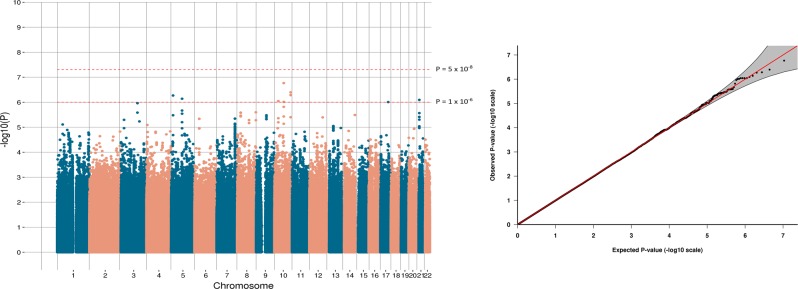
Manhattan and Q-Q plots of the GWAS of antidepressant stages of resistance. Genome-wide significance level (*P* < 5 × 10^−8^) is represented by a red line and suggestive threshold (*P* < 1 × 10^−5^) is represented by a blue line

In the GWAS of stages of antidepressant resistance in GS:SFHS (*n* = 3452), no SNP reached genome-wide significance (*P* > 5 × 10^−8^). The most significant SNP identified was an intergenic variant located at 10q22.1 (lead SNP rs116902282, *P* = 1.5 × 10^−7^, beta = 0.49, s.e. = 0.076; Fig. 2).

The top four loci for each GWAS below a *P*-value of 5 × 10^−7^ for the antidepressant treatment resistance meta-analysis and stages of antidepressant resistance can be found in Table 1. Heterogeneity statistics for the treatment resistance meta-analysis are also reported, we found either no evidence or nominal evidence of heterogeneity between GS:SFHS and GENDEP that was not significant after adjustment for multiple correction at a genome-wide level.

**Table 1:** Top GWAS loci in antidepressant treatment resistance and stages of resistance

Treatment resistance (meta-analysis)											
SNP	Chr	*P*-value	OR (CI)	Average freq	Alleles	Locus	Gene or (closest)	GS:SFHS OR (CI)	GENDEP OR (CI)	Heterogeneity *I*^2^	Heterogeneity *P-*value
rs188352979	10	3.3 × 10^-7^	2.87 (2.47–3.28)	0.017	A/G	Intergenic	(ACADSB–HMX3)	3.33 (2.90–3.76)	1.33 (0.31–2.35)	62.0	0.10
rs145842949	16	3.9 × 10^-7^	2.42 (2.08–2.77)	0.030	T/G	Intergenic	(MON1B)	2.35 (1.96–2.74)	2.69 (1.96–3.42)	0.0	0.75
rs111968111	6	3.9 × 10^-7^	3.00 (2.58–3.42)	0.014	T/C	Intergenic	(SRPK1–SLC26A8)	3.53 (3.08–3.98)	0.84 (-0.42–2.10)	77.5	0.035
rs138583130	12	5.0 × 10^-7^	2.50 (0.1894)	0.023	A/C	Intergenic	(CNTN1)	2.57 (2.16–2.98)	2.72 (1.75–3.68)	0.0	0.92

Stages of resistance (GS:SFHS)											
SNP	Chr	*P*-value	Beta (SE)	Freq	Alleles	Locus	Gene or (closest)				
rs116902282	10	1.5 × 10^-7^	0.49 (0.076)	0.011	T/C	Intergenic	(C10orf35–COL13A1)				
rs188352979	10	4.0 × 10^-7^	0.33 (0.065)	0.016	A/G	Intergenic	(ACADSB–HMX3)				
rs182041872	10	5.2 × 10^-7^	0.30 (0.060)	0.019	G/A	Intergenic	(ACADSB–HMX3)				
rs4400118	5	5.4 × 10^-7^	0.29 (0.058)	0.020	G/A	Intergenic	(DNAH5–TRIO)				

*SNP* Single nucleotide polymorphism, *OR* odds ratio,*MAF* minor allele frequency,*CI* confidence interval,*SE* standard error, *GS:SFHS* Generation Scotland: Scottish Family Health Study, *GENDEP* Genome-based Therapeutic Drugs for Depression

### Gene and gene-set enrichment analysis

Gene-based analysis with MAGMA did not identify any genes significantly associated after false discovery rate (FDR) multiple testing correction. Similarly, in the gene-set analysis no gene-set passed multiple testing correction over 10,000 permutations. The most significant genes and gene sets are listed in Supplemental Table S5.

### Pedigree-based heritability and genetic correlations

Pedigree-based heritability was calculated in GS:SFHS at 0.60 (CI = 0.22–0.87) for antidepressant treatment resistance and 0.27 (CI = 0.24–0.31) for stages of antidepressant resistance.

Significant positive genetic correlations were found between antidepressant treatment resistance and neuroticism (*r*_g_ = 0.66, *P*_FDR_ = 0.0091), MDQ (*r*_g_ = 0.86, *P*_FDR_ = 0.0072) and GHQ (*r*_g_ = 0.96,*P*_FDR_ = 8.8 × 10^–5^). For stages of antidepressant resistance, they were identified between neuroticism (*r*_g_ = 0.51, *P*_FDR_ = 0.023), SPQ (*r*_g_ = 0.44, *P*_FDR_ = 0.036), MDQ (*r*_g_ = 0.69,*P*_FDR_ = 0.027) and GHQ (*r*_g_ = 0.71,*P*_FDR_ = 0.0011). All these correlations survived correction for multiple testing with FDR (Table 2).

**Table 2:** Genetic correlations of antidepressant treatment resistance and stages of resistance in Generation Scotland: Scottish Family Health Study with cognitive and personality traits

	Treatment resistance					Stages of resistance		
	*P*-value	*P*_FDR_	*r*_g_ (s.e.)	*N*	*P*-value	*P*_FDR_	*r*_g_ (s.e.)	*N*
Neuroticism	**0.0034**	**0.0091**	**0.66** **(0.26)**	**3133**	**0.0058**	**0.023**	**0.51** **(0.19)**	**3133**
Extraversion	0.78	0.78	−0.059 (0.22)	3133	0.71	0.71	−0.063 (0.17)	3133
SPQ	0.070	0.11	0.43 (0.26)	1607	**0.018**	**0.036**	**0.44** **(0.20)**	**1607**
MDQ	**0.0018**	**0.0072**	**0.86** **(0.36)**	**1715**	**0.010**	**0.027**	**0.69** **(0.28)**	**1715**
GHQ	**1.1** **×** **10**^**−5**^	**8.8** **×** **10**^**−5**^	**0.96** **(0.26)**	**3378**	**0.00014**	**0.0011**	**0.71** **(0.19)**	**3378**
‘*g*’	0.32	0.37	−0.16 (0.17)	3349	0.067	0.091	−0.24 (0.133)	3349
Education	0.13	0.17	−0.33 (0.23)	3233	0.40	0.46	−0.15 (0.18)	3233
SIMD	0.063	0.11	−0.22 (013)	3268	0.068	0.091	−0.18 (0.099)	3268

Significant values after multiple testing correction (*P*_FDR_ < 0.05) are shown in bold

*FDR* False discovery rate,*rP* phenotypic correlation,*SPQ* schizotypal personality questionnaire, *MDQ* mood disorder questionnaire, *GHQ* general health questionnaire, *SIMD* Scottish index of multiple deprivation

### Genetic liability of psychiatric traits with antidepressant treatment resistance and stages of resistance using polygenic risk scores techniques

Antidepressant treatment resistance and stages of antidepressant resistance were positively and nominally associated with MDD PRS at *P*-value thresholds (*P*_T_) 0.1, 0.5 and 1, and antidepressant treatment resistance only with schizophrenia PRS at *P*_T_ < 0.01. There were no significant associations between antidepressant treatment resistance or stages of antidepressant resistance with bipolar disorder PRS (Table 3). No result survived FDR correction and power analyses indicated we were underpowered to detect an association between MDD and bipolar disorder PRS with antidepressant treatment resistance and stages of antidepressant resistance. Schizophrenia PRS was powered to detect an association at all thresholds in both antidepressant treatment resistance and stages of resistance given a genetic correlation of 0.5. At a genetic correlation of 0.25, stages of antidepressant resistance had adequate power at all thresholds whilst antidepressant treatment resistance was only powered at*P*_T_ 0.01 (Table 3).

**Table 3:** PRS associations with schizophrenia, bipolar disorder and MDD with antidepressant treatment resistance and stages of resistance

Treatment resistance				Stages of resistance		
Threshold	*P-*value (*P*_FDR_)	Stats	Power at *r*_g_ 0.5 (& 0.25)	*P-*value (*P*_FDR_)	Stats	Power at *r*_g_ 0.5 (& 0.25)
MDD						
0.01	0.35	*β* = 0.25	0.17	0.45	*β* = 0.0055	0.44
	(0.54)	*R*^2^ = 0.00030	(0.079)	(0.57)	*R*^2^ = 0.00021	(0.15)
0.05	0.22	*β* = 0.0058	0.17	0.21	*β* = 0.0086	0.45
	(0.46)	*R*^2^ = 0.00050	(0.080)	(0.46)	*R*^2^ = 0.00051	(0.15)
0.1	0.042	*β* = 0.0017	0.17	0.049	*β* = 0.014	0.44
	(0.21)	*R*^2^ = 0.0014	(0.079)	(0.25)	*R*^2^ = 0.0013	(0.15)
0.5	0.012	*β* = 0.012	0.16	0.015	*β* = 0.017	0.41
	(0.20)	*R*^2^ = 0.0020	(0.076)	(0.15)	*R*^2^ = 0.0020	(0.14)
1	0.030	*β* = 0.060	0.16	0.020	*β* = 0.0160	0.40
	(0.20)	*R*^2^ = 0.0015	(0.076)	(0.15)	*R*^2^ = 0.0018	(0.14)
SCZ						
0.01	0.027	*β* = 0.011	1.0	0.14	*β* = 0.010	1.0
	(0.20)	*R*^2^ = 0.0017	(0.85)	(0.36)	*R*^2^ = 0.00074	(1.0)
0.05	0.19	*β* = 0.0061	1.0	0.64	*β* = 0.0032	1.0
	(0.46)	*R*^2^ = 0.00055	(0.75)	(0.74)	*R*^2^ = 7.1 × 10^−5^	(1.0)
0.1	0.68	*β* = 0.0019	1.0	0.87	*β* = 0.0011	1.0
	(0.68)	*R*^2^ = 5.3 × 10^−5^	(0.68)	(0.87)	*R*^2^ = 8.1 × 10^-6^	(0.99)
0.5	0.40	*β* = 0.0038	0.98	0.48	*β* = 0.0047	1.0
	(0.56)	*R*^2^ = 0.00021	(0.52)	(0.60)	*R*^2^ = 0.00015	(0.95)
1	0.42	*β* = 0.0036	0.98	0.45	*β* = 0.0050	1.0
	(0.56)	*R*^2^ = 0.00020	(0.50)	(0.60)	*R*^2^ = 0.00018	(0.94)
BPD						
0.01	0.57	*β* = −0.0025	0.25	0.70	*β* = 0.0026	0.64
	(0.65)	*R*^2^ = 9.6 × 10^−5^	(0.099)	(0.75)	*R*^2^ = 4.7 × 10^−5^	(0.21)
0.05	0.59	*β* = 0.0024	0.26	0.45	*β* = 0.0050	0.65
	(0.65)	*R*^2^ = 8.6 × 10^−5^	(0.10)	(0.60)	*R*^2^ = 0.00017	(0.22)
0.1	0.45	*β* = 0.0034	0.25	0.26	*β* = 0.0074	0.65
	(0.56)	*R*^2^ = 0.00017	(0.099)	(0.48)	*R*^2^ = 0.00038	(0.22)
0.5	0.23	*β* = 0.0053	0.24	0.11	*β* = 0.010	0.61
	(0.46)	*R*^2^ = 0.00042	(0.095)	(0.36)	*R*^2^ = 0.00074	(0.20)
1	0.23	*β* = 0.0053	0.23	0.13	*β* = 0.0097	0.60
	(0.46)	*R*^2^ = 0.00041	(0.095)	(0.36)	*R*^2^ = 0.00066	(0.20)

*MDD* Major depressive disorder, *SCZ* schizophrenia,*BPD* bipolar disorder,*r*_*g*_ genetic correlation

## Discussion

We utilised antidepressant prescription records to explore common genetic factors in antidepressant treatment resistance and stages of resistance in a population and family-based cohort of 3452 individuals. In the treatment resistance GWAS meta-analysis, the most significant locus was located at 10q26.13 at *P* = 3.3 × 10^−7^; lead SNP rs188352979. This SNP is intergenic and lies in between genes *ACADSB* and *HMX3*.*ACADSB* encodes short/branched chain specific acyl-CoA dehydrogenase (SBCAD) which is an enzyme involved in the metabolism of fatty acids [51]. Of interest, differences in mitochondrial fatty acid metabolism have been found between ketamine responders and non-responders in bipolar disorder [52]. *HMX3* is a transcription factor that is involved in the specification of neuronal cells needed for hypothalamus development and hypothalamic–pituitary–adrenal (HPA) axis [53]. Disruptions in the HPA axis are known to be associated with MDD, MDD severity and antidepressant response [55, 56]. A single locus at 10q22.1 was associated with stages of antidepressant resistance at *P* = 1.71 × 10^−7^, lead SNP rs116902282. This is an intergenic variant that lies between functional genes *C10orf35* and *COL13A1*. *C10orf35* is a protein coding gene that has previously been associated with uterine leiomyoma [56]. *COL13A1* encodes the alpha chain of one of the nonfibrillar collagens. No variant in either analysis reached the required threshold for genome-wide statistical significance (*P* > 5 × 10^−8^). Gene and gene-set enrichment did not identify any significant associations with either antidepressant treatment resistance or stages of resistance. Nonetheless, modest to high pedigree-based heritability estimates indicate that 60% of the variance in antidepressant treatment resistance and 27% of the variance in stages of resistance can be explained by genetics, although these estimates had large confidence intervals. This indicates that further exploration into genetic contributions in antidepressant resistance is warranted.

Antidepressant treatment resistance was significantly and positively genetically correlated with neuroticism (*r*_g_ = 0.66), MDQ (*r*_g_ = 0.86) and GHQ (*r*_g_ = 0.96) indicating overlapping genetic architecture between these traits. In stages of antidepressant resistance, these same traits also demonstrated significant genetic correlation; neuroticism (*r*_g_ = 0.51), MDQ (*r*_g_ = 0.69) and GHQ (*r*_g_ = 0.71), and we additionally identified a significant genetic correlation with SPQ (*r*_g_ = 0.44). We consider all correlations over 0.5 to be high genetic correlation (as defined previously [57]) and therefore only identify one correlation not considered to be so. The MDQ and SPQ correlation indicates that more resistant individuals may share genetic components with schizotypy and mood disorder personality traits. Genetic overlap between psychological distress and antidepressant resistance is indicative that individuals susceptible to distress are associated with a poorer outcome in antidepressants and should be further investigated. Nevertheless, only a modest correlation was identified between stages of antidepressant resistance and SPQ and it is difficult to ascertain causal inferences with any of these correlations. Moreover, we did not find any correlation in antidepressant treatment resistance or stages of resistance with general intelligence, education or social deprivation traits, however, a lack of power due to small sample size (*n* = 3452 with only 250 treatment resistant cases) may have been major contributing factor.

Using PRS, we investigated whether poor response to antidepressants indicate a higher liability to other mental disorders (schizophrenia or bipolar disorder) as well as higher genetic loading of MDD itself. We did not find any significant association with antidepressant treatment resistance or stages of resistance, however, power with the current sample size was only adequate in analysis with schizophrenia PRS at a genetic correlation of 0.5 and additionally 0.25 for stages of antidepressant resistance. Results for MDD and bipolar disorder PRS should therefore be treated with caution, although the nominal associations between MDD PRS and stages of antidepressant resistance may be worth further investigation with larger sample sizes.

Interindividual variability in drug therapies can often be attributed to genetic variability in cytochrome P450s (CYP450) and metabolic transporters. In this study, we did not identify any associations with metabolic variants and response but it has been widely explored in previous literature, although inconsistent results have been reported for both *CYP450*s [58] and metabolic transporters [59]. For instance, nortriptyline has been widely demonstrated to show differential serum level per *CYP2D6* metaboliser type (reviewed previously [58]), however a GENDEP study of 223 individuals taking escitalopram and 161 individuals taking nortriptyline found that whilst serum levels of the drugs varied per *CYP450* genotype, treatment response was not affected [60]. Nevertheless, it has been found that *CYP2D6* poor metabolisers have a higher proportion of side effects [61]. Venlafaxine, however, has been more consistently shown to have lower response and remission rates amongst *CYP2D6* poor metabolisers [62, 63]. This indicates that, given access to larger samples, studies exploring individual antidepressants may be beneficial to identify metabolic profiles that may or may not be affecting response. Nonetheless, no GWAS to our knowledge has so far implicated *CYP450* in association with treatment response.

One of the strengths of this study was that it used data from a population-based cohort and is a good representation of antidepressant users in a MDD sample in the general Scottish population. Nevertheless, certain limitations of this study should be noted. Although currently diagnosed schizophrenia and bipolar patients were removed prior to this analysis and minimum dose was matched to that of MDD recommendations, it is possible that individuals were prescribed the antidepressant for other conditions, e.g. anxiety disorders, obsessive compulsive disorder (OCD), posttraumatic stress disorder (PTSD) and panic disorders, and may have had a misdiagnosis in the first instance. Nevertheless, the high genetic correlation between all antidepressant users and SCID-diagnosed MDD demonstrates that using prescription records with dose and duration exclusions may be a valid method of identifying large samples of proxy MDD cases that are genetically representative of the disorder. It was not possible to account for the use of psychotherapy or ECT (which is advised in patients with severe MDD) and, additionally, no exclusions were applied for the prescription of other psychotropic medications used alongside antidepressants. It is therefore possible that other treatments may have provided more information of response and non-response to antidepressants. We were also only able to obtain prescription records over a 6-year period meaning there are likely to be some individuals who had prescriptions before this period. Despite the 6-week threshold applied here, individuals may also switch antidepressants after 6 weeks continuous treatment, due to side effects such as weight gain [64]. An additional limitation to this method is that it does not account for combination therapies. Although in the UK, combination therapies are often only considered after failure of a second single trial of antidepressant monotherapy [27], therefore by the definition adopted in this current study, they would also be considered treatment resistant. Furthermore, because we integrate prescribing data across a number of different antidepressant drugs and classes, specific associations with treatment resistance within or between classes of prescribed compounds may have been missed. It should also be highlighted that, whilst antidepressant resistance measures were taken over a 6-year period, mood and personality questionnaires were taken at a single time point and may have been impacted by mood or condition (e.g. currently having a depressive episode) of the patient at the time of completing the questionnaire. Lastly, differing covariates were applied in the meta-analyses, e.g. baseline severity in the GENDEP sample but not GS:SFHS.

Whilst the stages of antidepressant resistance measure provide additional information above the binary treatment resistance measure, it differs from models previously defined in the literature. Previous measures (such as the Thase and Rush five-stage model [65], Massachusetts General Hospital staging model [29] and the Maudsley staging model [66]) are significantly more detailed and account for augmentation therapies, ECT, differing antidepressant classes and MDD severity. However, there has  also been criticism of these measures as they have arbitrary thresholds and have not been widely validated [67]. In this study, our staging measure was defined solely on antidepressant switching, due to limitations in the available data. This measure is therefore not synonymous with other definitions used in prior studies. Nevertheless, there is no uniformly accepted method of quantifying the degree of antidepressant resistance and this measure will likely capture useful information regarding resistance to antidepressant treatment.

Despite an increased sample size compared to those reported in previous clinical studies of antidepressant response (*n* = 2897), our numbers are still small for a GWAS and it is likely that we were underpowered. With an increasing availability of electronic records in large biobanks and numerous smaller antidepressant studies, a collaborative effort approach may be required in order to increase sample size for adequate power. To replicate our analysis with adequate power (>0.8) at a MAF of 0.01, it would require a sample size of 7596 cases for antidepressant treatment resistance and 9660 total sample for stages of resistance assuming an OR of 1.6 and beta of 0.3, respectively (Supplemental Table S6; power calculations were completed using QUANTO v1.2). Furthermore, the epigenetic of treatment resistance could be further explored. Recent studies have indicated that methylation levels have been predictive of overall response [68].

With increasing accessibility of electronic health records [69], access to prescription records is becoming possible. In this study, we explored the possibility of utilising this prescription data to examine resistance to antidepressant treatment by inferring drug switching as non-response. We have provided evidence that resistant individuals have a high genetic correlation with neuroticism, psychological distress, schizotypy and mood disorder traits. Furthermore, we demonstrate the need for larger cohorts and collaboration in order to maximise sample size. This study demonstrates the value of this method and,  as larger cohort sizes become available, the results of such studies could further inform clinical and research applications.

## Disclaimer

This report represents independent research part-funded by the National Institute for Health Research (NIHR) Biomedical Research Centre at South London and Maudsley NHS Foundation Trust and King’s College London. The views expressed are those of the authors and not necessarily those of the NHS, the NIHR, or the Department of Health.

## Supplementary information

Supplementary Materials
