# Supplementary material for: Genome-wide association study of antidepressant treatment resistance in a population-based cohort using health service prescription data and meta-analysis with GENDEP
Source: Pharmacogenomics J. 2019 Jan 31;20(2):329–41. doi: 10.1038/s41397-019-0067-3 (PMC7096334; doi:10.1038/s41397-019-0067-3)
Supplement: Supplementary file 1 — Supplementary Materials [file 41397_2019_67_MOESM1_ESM.docx]

**This document contains the supplementary materials for the manuscript by Wigmore et al “Genome-wide association study of antidepressant treatment resistance in a population-based cohort using health service prescription data with meta-analysis with GENDEP.”**

**Supplemental Methods**

**Cohort description**

**Generation Scotland: Scottish Family Health Study (GS:SFHS):** GS:SFHS is a cohort of 24,080 participants, aged between 18 and 98 (mean age=47.6, s.d.=15.4), recruited from Scottish medical practices between 2006 and 2011. Participants were recruited at random after individuals were identified via their Community Health Number and it was a requirement for participants to also have a first-degree relative over the age of 18 willing to participate. Data on mood, cognition and personality were obtained through questionnaires and genotype data was obtained for 20,032 individuals (after QC n=19,994). Schizophrenia and bipolar individuals were identified through record linkage to the Scottish Morbidity Record, general hospital inpatient, general hospital outpatient and psychiatric inpatient/outpatient data (SMR00, SMR01, SMR04). Prescription data was available through the Prescribing Information System (PIS) which records all Scottish NHS prescriptions for payments for medications prescribed by general practitioners (GPs), nurses, pharmacists and hospitals where the medication was dispensed in the community (hospital dispensed prescriptions are not included). Records were available for the period between April 2009 and February 2015.

**SCID-Diagnosed MDD**

MDD in GS:SFHS was diagnosed by structured clinical interview for DSM-IV (SCID) if individuals screened positive during initial interview. 2,643 individuals were diagnosed with MDD (controls=17,119) and bipolar patients were removed (n=76).

**Prescription records in GS:SFHS**

Information available from prescription records included the dose of the prescription given, amount of drug dispensed and prescription instructions. From this, a daily dose was calculated based on the dose of the prescription and the instructions for daily intake. Prescription instructions were sometimes given as a range, where this was the case, e.g. “1-2 tablets per day”, the lower range was selected to calculate daily dose to ensure an individual was taking at least an adequate dose. For calculations regarding duration of prescription, the upper range was selected to ensure an adequate duration. Duration on the antidepressant was calculated based on the dispensed amount and daily dose. Entries were then distinguished into prescription episodes. i.e. periods where the same antidepressant had been prescribed continuously for at least 6 weeks. All prescription episodes that fell below this were excluded. Duration of a drug was calculated additively based on a continuous prescription. Date of drug dispense was available at month and year only therefore when determining a continuous prescription time point a 31-day margin was given. British National Formulary (BNF) guidelines differ for elderly patients (age>64 years) therefore age at prescription was considered when excluding those below the minimum dose (a total of 29,942 entries excluded). BNF guidelines recommend that at least 4 weeks of treatment should be completed before considering switching the antidepressant due to lack of efficacy however we applied a stricter threshold of at least 6 weeks.

**Cognitive and personality traits in GS:SFHS**

To obtain ‘g’ (general intelligence) a principle component analysis (PCA) was conducted based on each of the component cognitive test scores. 4 Cognitive tests were assessed: digit symbol coding, vocabulary, verbal fluency and logical memory. Digit symbol coding was measured using the Wechsler digit symbol substitution task^4^ to measure participants processing speed. For vocabulary, the Mill Hill vocabulary scale^5^ (combined junior and senior synonyms) was used and, to test executive functioning, a 1 minute letter-based verbal fluency test was used with letters C, F and L^6^. Logical memory was assessed by summation of the immediate and delayed score for recall of a paragraph in the Wechsler logical memory test^4^. The first unrotated principle component was utilised as ‘g’ and explained 41% of the total variance between the 4 tests.

Neuroticism and extraversion were assessed using the Eysenck Personality Questionnaire Short Form-Revised^7^ with a score ranging 0-12. Schizotypal personality and mood disorders were measured by the schizotypal personality questionnaire (SPQ) version B^8^ and mood disorder questionnaire (MDQ)^9^, respectively. Higher scores indicated higher expression of the personality trait. The general health questionnaire 28 (GHQ)^10^ was used as a measure of psychological distress with 28 questions scored from 0 (“not at all”) to 3 (“much more than usual”).

To measure social deprivation, the Scottish Index of Multiple Deprivation (SIMD)^11^ was used. In brief, this ranks areas according to crime, housing, education, income, health and geographical access. Each area then receives a rank from 1 to 6,505 which is then converted to a quintile, 1 being most deprived and 5 being least deprived. Education was scored as years in full-time education and ranged from 0-10.

**Construction of Genetic Relationship Matrices (GRMs)**

Two GRMs were fitted utilising the method created by Zaitlen *et al.* (2013) The first GRM included pairwise relationship coefficients for all individuals and the second included off-diagonal elements of pairs of individuals who had a relationship coefficient < 0.05 set to 0. This therefore excluded pairs of individuals that have a most recent common ancestor of approximately four generations distant, assuming no inbreeding. This method has been demonstrated to account for potential upward biases due to excessive relationships, thus allowing the inclusion of closely and distantly related individuals in genetic analyses^12^.

**Taylor series transformation**

The Taylor series transformation^13^ is used to convert a linear beta to an odds ratio (OR) using the formula $OR= \frac{P+beta/(1-P-beta)}{P/(1-P)}$, where P indicates the trait prevalence in the general population and beta is the beta coefficient from the linear association model.

**Gene and gene-set enrichment using MAGMA**

MAGMA^14^ uses multiple regression to identify gene-sets that are associated with the phenotype while accounting for linkage disequilibrium (LD) between markers by combing SNP *P* values. The LD reference panel used was the European-ancestry subjects from 1000 Genomes Project^15^. Both individual level genotype data and summary statistics can be used as input, however individual level genotype data are considered the preferred choice.

**Polygenic risk scores (PRS)**

PRS is an additive SNP score constructed per individual weighted by the effect size from a training set (GWAS summary statistics). Due to the application of linkage disequilibrium (LD) pruning, PRS were constructed utilising genotyped data rather than imputed. Data was quality controlled (QC) using the following thresholds; minor allele frequency (MAF) > 1%, Hardy Weinberg equilibrium (HWE) p>1x10^-6^, missingness per individual <1%, missingness per SNP <1%. Clump based pruning was used (r^2^=0.25, 300kb window) and strand-ambiguous SNPs were removed. Multi-dimensional scaling (MDS) components were created in PLINK^17^ to control for population stratification. Further details on this method can be found in the original paper^18^.

Trait variance explained by the PRS was calculated using (var(*x* × *β*))/var(*y*), where *x* was the standardized PGS, *β* was the corresponding regression coefficient and *y* was the phenotype^19^

**Psychiatric Genomics Consortium (PGC) MDD GWAS summary statistics**

Unpublished data was used to construct PRS in GS:SFHS using the latest PGC release for MDD. The sample consisted of 51,865 MDD cases and 112,200 controls.

**Genetic Correlation**

Genetic correlations (*r_g_*) is defined as $r_{g}= \frac{{cov}_{G}}{\sqrt{V_{Gi}{*V}_{Gj}}}$ where cov_G_ is the additive covariance, V_Gi_ is the additive variance of the personality or cognitive variable and V_Gj_ is the additive variance of treatment resistance or stages of resistance. Significance was calculated using the likelihood ratio test to compare the model against a null model (model assuming no covariance between the traits).

**References**

1. Fava M. Diagnosis and definition of treatment-resistant depression. *Biol Psychiatry* 2003; **53**(8)**:** 649-659.

2. Hazari H, Christmas D, Matthews K. The clinical utility of different quantitative methods for measuring treatment resistance in major depression. *J Affect Disord* 2013; **150**(2)**:** 231-236.

3. Berlim M, Turecki G. Definition, Assessment, and Staging of Treatment—Resistant Refractory Major Depression: A Review of Current Concepts and Methods. *Can J Psychiatry* 2007; **52**(1)**:** 46-54.

4. Wechsler D. WAIS-III UK Administration and Scoring Manual. *London* 1998; **Psychological Corporation**.

5. Raven J, Raven J, Court J. Manual for Raven’s progressive matrices and vocabulary scales. *London* 1998; **HK Lewis**.

6. Lezak M. Neuropsychological Assessment. *New York* 1995; **Oxford University Press**.

7. Eysenck H. Dimensions of personality: 16, 5 or 3 criteria for a taxonomic paradigm. *Pers Individual Differ* 1991; **12**(8)**:** 773-790.

8. Raine A, Benishay D. The SPQ-B: A Brief Screening Instrument for Schizotypal Personality Disorder. *J Pers Disord* 1995; **9:** 346-355.

9. Hirschfeld RM. The Mood Disorder Questionnaire: A Simple, Patient-Rated Screening Instrument for Bipolar Disorder. *Prim Care Companion J Clin Psychiatry* 2002; **4**(1)**:** 9-11.

10. Goldberg DP, Hillier VF. A scaled version of the General Health Questionnaire. *Psychol Med* 1979; **9**(1)**:** 139-145.

11. Payne R, G A. UK indices of multiple deprivation – a way to make comparisons across constituent countries easier. *Health Stat Q* 2012; **22**.

12. Zaitlen N, Kraft P, Patterson N, Pasaniuc B, Bhatia G, Pollack S*, et al*. Using extended genealogy to estimate components of heritability for 23 quantitative and dichotomous traits. *PLoS Genet* 2013; **9**(5)**:** e1003520.

13. Cortes A, Hadler J, Pointon JP, Robinson PC, Karaderi T, Leo P*, et al*. Identification of multiple risk variants for ankylosing spondylitis through high-density genotyping of immune-related loci. *Nat Genet* 2013; **45**(7)**:** 730-738.

14. de Leeuw CA, Mooij JM, Heskes T, Posthuma D. MAGMA: generalized gene-set analysis of GWAS data. *PLoS Comput Biol* 2015; **11**(4)**:** e1004219.

15. Abecasis GR, Auton A, Brooks LD, DePristo MA, Durbin RM, Handsaker RE*, et al*. An integrated map of genetic variation from 1,092 human genomes. *Nature* 2012; **491**(7422)**:** 56-65.

16. Zheng J, Erzurumluoglu AM, Elsworth BL, Kemp JP, Howe L, Haycock PC*, et al*. LD Hub: a centralized database and web interface to perform LD score regression that maximizes the potential of summary level GWAS data for SNP heritability and genetic correlation analysis. *Bioinformatics* 2017; **33**(2)**:** 272-279.

17. Purcell S, Neale B, Todd-Brown K, Thomas L, Ferreira M, Bender D*, et al*. PLINK: a tool set for whole-genome association and population-based linkage analyses. *Am J Hum Genet* 2007; **81**(3)**:** 559-575.

18. International Schizophrenia Consortium, Purcell SM, Wray NR, Stone JL, Visscher PM, O'Donovan MC*, et al*. Common polygenic variation contributes to risk of schizophrenia and bipolar disorder. *Nature* 2009; **460**(7256)**:** 748-752.

19. Nakagawa S, Schielzeth H. A general and simple method for obtaining *R^2^* from generalized linear mixed-effects models. *Methods Ecol Evol* 2013; **4**(2)**:** 133-142.

**Supplemental Table S1. Full list of antidepressants being prescribed in GS:SFHS.**

| **Antidepressant Class** | **Prescription name** | **Frequency** |
| --- | --- | --- |
| *Selective Serotonin Reuptake Inhibitors (SSRI)* | CITALOPRAM | 1555 |
|  | ESCITALOPRAM | 117 |
|  | FLUOXETINE | 1130 |
|  | FLUVOXAMINE MALEATE | 2 |
|  | PAROXETINE | 105 |
|  | SERTRALINE | 584 |
|  | **Total** | **3493** |
| *Serotonin Noradrenaline Reuptake Inhibitors (SNRI)* | DULOXETINE | 114 |
|  | VENLAFAXINE | 224 |
|  | **Total** | **338** |
| *Serotonin Antagonist and Reuptake Inhibitors (SARI)* | TRAZODONE HYDROCHLORIDE | 66 |
|  | **Total** | **66** |
| *Noradrenaline Reuptake Inhibitors (NRI)* | REBOXETINE | 4 |
|  | **Total** | **4** |
| *Tricyclic Antidepressants (TCA)* | AMITRIPTYLINE | 178 |
|  | CLOMIPRAMINE HYDROCHLORIDE | 25 |
|  | DOSULEPIN HYDROCHLORIDE | 48 |
|  | DOXEPIN | 5 |
|  | IMIPRAMINE HYDROCHLORIDE | 14 |
|  | LOFEPRAMINE | 39 |
|  | NORTRIPTYLINE | 7 |
|  | TRIMIPRAMINE | 3 |
|  | **Total** | **319** |
| *Tetracyclic Antidepressants (TeCA)* | MIRTAZAPINE | 453 |
|  | **Total** | **453** |
| *Monoamine Oxidase Inhibitors (MAOI)* | MOCLOBEMIDE | 6 |
|  | PHENELZINE | 6 |
|  | TRANYLCYPROMINE | 3 |
|  | **Total** | **15** |
| *Melatonergic Antidepressants* | AGOMELATINE | 2 |
|  | **Total** | **2** |

**Supplemental Table S2. Description of GS:SFHS subjects included in TR study.**

| **Variable** | **TR (n=250)** | **Non-TR (n=3,202)** |
| --- | --- | --- |
| Age | 45.04±13.96 | 47.32±13.92 |
| Gender (F/M) | 195/55 | 2335/867 |
| Total antidepressants taken | 3 for 186 individuals (67.2%)  4 for 46 individuals (18.4%)  5 for 13 individuals (5.2%)  6 for 1 individuals (0.4%)  7 for 2 individuals (0.8%)  8 for 2 individuals (0.8%) | 1 for 2557 individuals (79.9%)  2 for 645 individuals (20.1%) |
| N of previous SSRI treatments | 0 for 8 individuals (3.2%)  1 for 53 individuals (21.2%)  2 for 118 individuals (47.2%)  3 for 69 individuals (27.6%)  4 for 2 individuals (0.8%) | 0 for 539 individuals (16.8%)  1 for 2333 individuals (72.9%)  2 for 330 individuals (10.3%) |
| N of previous TCA treatments | 0 for 200 individuals (80%)  1 for 41 individuals (16.4%)  2 for 9 individuals (3.6%) | 0 for 2993 individuals (93.5%)  1 for 206 individuals (6.4%)  2 for 3 individuals (0.1%) |
| N of previous MAOI antidepressants | 0 for 244 individuals (97.6%)  1 for 6 individuals (2.4%) | 0 for 3193 individuals (99.7%)  1 for 9 individuals (2.8%) |
| N of previous treatments with other antidepressants | 0 for 202 individuals (80.8%)  1 for 139 individuals (55.6%)  2 for 51 individuals (20.4%)  3 for 12 individuals (4.8%) | 0 for 2645 individuals (82.6%)  1 for 524 individuals (16.4%)  2 for 33 individuals (1.0%) |

**Supplemental Table S3. Description of GENDEP subjects included in TR study.**

| **Variable** | **TR (n=109)** | **Non-TR (n=668)** |
| --- | --- | --- |
| Age | 43.54±11.15 | 41.90±11.62 |
| Gender (F/M) | 73/36 | 411/257 |
| Baseline MADRS | 30.38±6.62 | 28.58±6.74 |
| Baseline HAMD | 23.03±5.01 | 21.66±5.26 |
| Age at onset | 32.10±11.88 | 31.86±10.38 |
| N of previous episodes | 1.95±0.74 | 1.69±0.66 |
| Antidepressant during longitudinal trial | Escitalopram: 50  Nortriptyline: 59 | Escitalopram: 391  Nortriptyline: 277 |
| Antidepressant switch during longitudinal trial (yes/no) | 71/38 | 35/633 |
| N of previous SSRI treatments | 0 for 50 individuals (45.9%)  1 for 39 individuals (35.8%)  2 for 17 individuals (15.6%)  3 for 3 individuals (2.8%) | 0 for 498 individuals (74.6%)  1 for 145 individuals (21.7%)  2 for 19 individuals (2.8%)  3 for 6 individuals (0.9%) |
| N of previous TCA treatments | 0 for 68 individuals (62.4%)  1 for 28 individuals (25.7%)  2 for 10 individuals (9.2%)  3 for 3 individuals (2.8%) | 0 for 600 individuals (89.8%)  1 for 58 individuals (8.9%)  2 for 6 individuals (0.9%)  3 for 3 individuals (0.4%)  5 for 1 individuals (0.1%) |
| N of previous treatments with dual antidepressants | 0 for 89 individuals (81.7%)  1 for 19 individuals (17.4%)  2 for 1 individuals (0.9%) | 0 for 637 individuals (95.4%)  1 for 31 individuals (4.6%) |
| N of previous MAOI treatments | 0 for 104 individuals (95.4%)  1 for 5 individuals (4.6%) | 0 for 656 individuals (98.2%)  1 for 12 individuals (1.8%) |
| N of previous treatments with other antidepressants | 0 for 86 individuals (78.9%)  1 for 20 individuals (18.3%)  2 for 3 individuals (2.8%) | 0 for 635 individuals (95.1%)  1 for 31 individuals (4.6%)  2 for 2 individuals (0.3%) |
| Previous antidepressant trial yes/no | 77/32 | 281/387 |

**Supplemental Table S4. Genetic correlations between all antidepressant users and MDD.**

|  | **All Antidepressant users and MDD** | | |
| --- | --- | --- | --- |
|  | ***P* value** | ***r_g_*** | **SE** |
| Genetics (G) | 0.026 | 1.0 | 0.10 |
| Kinship (K) | 1.7x10^-16^ | 0.88 | 0.039 |

Abbreviations: MDD, major depressive disorder; *r_g_*, genetic correlation; SE, standard error

**Supplemental Table S5. Top MAGMA genes and gene-sets in treatment resistance and stages of resistance to antidepressants**

| **GENE ENRICHMENT ANALYSIS** | | | | | | | |
| --- | --- | --- | --- | --- | --- | --- | --- |
| **Treatment Resistance** | | | | **Stages of Resistance** | | | |
| **Genes** | ***P* value** | ***P*_FDR_** | **Effect Size** | **Genes** | ***P* value** | ***P*_FDR_** | **RSQ** |
| AASDHPPT | 5.81x10^-5^ | 0.465 | 8.0 | ZFP28 | 3.1x10^-5^ | 0.45 | 0.0082 |
| PLOD2 | 1.16x10^-4^ | 0.464 | 12.5 | ZNF600 | 1.7x10^-4^ | 0.54 | 0.0058 |
| NEUROG3 | 1.31x10^-4^ | 0.465 | 2.0 | ZFR2 | 4.5x10^-4^ | 0.51 | 0.0076 |
| **GENE-SET ENRICHMENT ANALYSIS** | | | | | | | |
| **Treatment Resistance** | | | | **Stages of Resistance** | | | |
| **Gene-sets** | ***P* value** | **Corrected *P*** | **Beta** | **Gene-sets** | ***P* value** | **Corrected *P*** | **Beta** |
| Sarcoplasmic reticulum calcium ion transport | 0.000162 | 0.334 | 0.929 | Innate immune response in mucosa | 0.00142 | 0.314 | 0.796 |
| Regulation of protein oligomerisation | 0.000184 | 0.370 | 0.581 | Necroptotic process | 0.000229 | 0.437 | 0.661 |
| Regulation of protein homo-oligomerisation | 0.000412 | 0.627 | 0.806 | Organ or tissue specific immune response | 0.00219 | 0.984 | 0.521 |

**Supplemental Table S6. Power analysis for GWAS of Treatment Resistance and Stages of Resistance for SNPs at a MAF of 1% and 5%**

Abbreviations: MAF, minor allele frequency; OR, odds ratio.

|  | **Treatment Resistance** | | **Stages of Resistance** | |
| --- | --- | --- | --- | --- |
| **MAF** | **OR** | **N cases** | **Beta** | **N** |
| *0.01* | 1.1  1.6  2.1 | 225,675  7,596  2,656 | 0.1  0.3  0.5 | 87,102  9,660  3,465 |
| *0.05* | 1.1  1.6  2.1 | 47,244  1,624  579 | 0.1  0.3  0.5 | 18,138  4,520  706 |
